# Case Report: Successful Implementation of Integrative Cognitive Remediation for Early Psychosis

**DOI:** 10.3389/fpsyt.2020.624091

**Published:** 2021-01-14

**Authors:** Olina G. Vidarsdottir, David L. Roberts, Elizabeth W. Twamley, Berglind Gudmundsdottir, Engilbert Sigurdsson, Brynja B. Magnusdottir

**Affiliations:** ^1^Department of Psychiatry, Landspitali—The National University Hospital, Reykjavik, Iceland; ^2^Faculty of Medicine, School of Health Sciences, University of Iceland, Reykjavik, Iceland; ^3^Division of Community Recovery, Research and Training, Department of Psychiatry, University of Texas Health Science Center, San Antonio, TX, United States; ^4^Department of Psychiatry, University of California, La Jolla, CA, United States; ^5^Center of Excellence for Stress and Mental Health and Research Service, VA San Diego Healthcare System, San Diego, CA, United States; ^6^Department of Psychology, Reykjavik University, Reykjavik, Iceland

**Keywords:** schizophrenia, functional outcome, social cognition and interaction training, compensatory cognitive training, rehabilitation

## Abstract

Many individuals demonstrate functionally relevant impairment in neurocognition as well as social cognition early on in the course of their psychotic disorder. There is robust evidence supporting cognitive remediation as an effective treatment of cognitive dysfunction in schizophrenia. Increasingly it is accepted that earlier treatment is associated with better outcome and that it is important to systematically assess and treat cognitive dysfunction before the cognitive and functional disabilities are fully realized. However, the clinical availability of these interventions remains sparse. As we move forward with implementing evidence-based interventions into multi-component treatment for early psychosis, it is important to reflect on experience as well as evidence. This case report aims to describe the implementation of an integrative cognitive remediation program in coordinated specialty care (CSC) for early psychosis in Iceland and investigate whether the intervention is sustainable in a CSC setting. Data on the number of patients treated, facilitators trained, groups conducted, and funding was used to assess the sustainability. The results show that since initial implementation in 2016, the intervention has been routinely available as part of standard care, with over 100 patients having received the treatment. The report discusses key factors in the successful implementation of the program.

## Introduction

Psychotic disorders are severe mental disorders that usually emerge in early adulthood, disrupting educational and employment opportunities which can result in a high rate of disability pensions (1). Neuro- and social-cognitive deficits are hallmark traits of psychotic disorders and have strong and consistent functional associations (2–6). Cognitive remediation (CR) is an evidence-based treatment for these cognitive impairments (7–9), and clinical practice guidelines published in countries around the world now recommend CR (10–12). However, clinical availability of CR remains sparse, resulting in an unsatisfactory gap between science and clinical practice (13).

It is generally accepted that earlier treatment of psychotic disorders is associated with better outcomes (14, 15). Therefore, the aim of early intervention in psychosis (EIP) services has been to minimize and shorten the severity of the first psychotic episode and facilitate recovery through early detection and intervention during the first 3–5 years following onset (16). The recommended setup for these EIP services is a multi-element program, known as Coordinated Specialty Care (CSC) that offers a range of evidence-based treatments (17). CSC programs differ from standard care in that a multidisciplinary team of mental health professionals provides evidence-based treatments that are tailored to the needs of each patient in a coordinated, integrated fashion instead of referring patients to different health care providers for each service. Evidence shows that synergistic pairing of psychosocial interventions and CR enhances the functional benefits of the intervention (8), making CSC programs especially attractive for implementation of CR. However, methods to assess and treat cognitive dysfunction are not a systematic part of CSC programs in countries around the world. Implementation research on CR is a relatively new field, but prior research indicates that CR can be successfully implemented in large-scale, geographically diverse, and publicly funded clinical settings (18). Although implementation models have been developed, investigations are needed into whether these models facilitate the implementation of CR in diverse settings. Describing different experiences with implementing CR is thus important.

In this report, we examine whether an integrated neuro- and social-cognitive remediation (ICR) program is sustainable in a CSC setting. More specifically, we sought to detail the implementation process and identify key factors contributing to successful implementation of ICR into the EIP service. We will delve into the case's implications for future service development and provide tips for success.

## Methods

**Figure 1:**
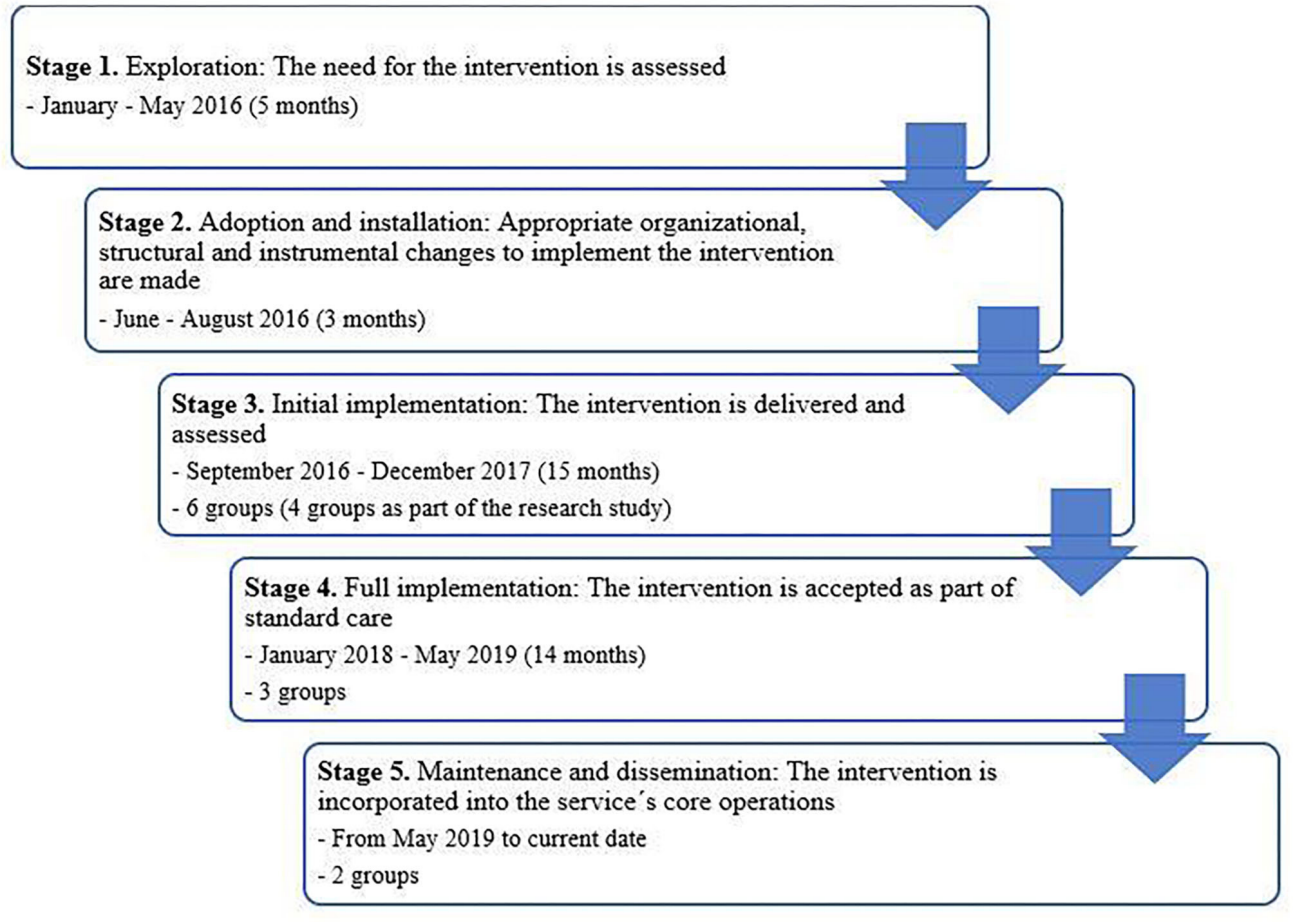
Timeline for the five stage of the implementation process.

The implementation process started in 2016. Figure 1 shows the timeline for implementation process. We describe the design and implementation in the five stages of CR implementation previously described and applied in other settings (19). To identify the key factors affecting the implementation of ICR, we administered a web-based survey to one consulting psychiatrist and three clinical directors directing the EIP service during the implementation process. They were asked to rate the importance of factors regarding the inner setting, adaptability, and relative effectiveness on a 5-point Likert-scale, with 1 = not important, 2 = slightly important, 3 = neutral, 4 = important, and 5 = very important. They also had the opportunity to comment on each question to further elaborate their answer. The procedures were deemed to be exempt from ethical review by the Landspitali—The National University Hospital's (LUH) ethical board.

### Setting and Population

The EIP service is part of LUH and is the only EIP service in Iceland. It is centralized in Reykjavik and serves the whole country, which has a population of around 367.000, with the majority (227.000) living in Reykjavik metropolitan area. The target population is individuals between 18-30 years old, experiencing their first episode of psychosis and are within five years of symptom onset. The service is intended for individuals whose acute psychotic symptoms have remitted or been stabilized, as well as those who continue to experience severe symptoms related to their first episode. Before being accepted into service, an ICD-10 diagnostic criteria for schizophrenia, schizotypal and delusional disorders (World Health Organization, 2008) is determined by an intake team of psychiatrists and other specialists in clinical adult psychology. Demographics as well as cognitive, clinical and functional outcomes of the patient population in service at the EIP have been described previously (5). The service is free of charge, with an inpatient (7 beds) and an outpatient service treating around 110 patients at any one time. It bases its care on a CSC program with a staff of 40 and of which 12 are case managers providing cognitive-behavioral case management. Upon entry, each patient receives care from a case manager, a supportive counselor, a psychiatrist, and a multidisciplinary team. All patients receive an individually based treatment including one or more of the following treatment components: family support, medication, psychoeducation, exercise, individual placement and support, and/or cognitive behavioral therapy for psychosis. No cognitive training was available prior to the implementation.

### Programmatic Elements

ICR was based on the following three cognitive remediation approaches: Neuropsychological Educational Approach to Remediation (NEAR) (20), Compensatory Cognitive Training (CCT) (21), and Social Cognition and Interaction Training (SCIT) (22). ICR program components have been described elsewhere (9), but are summarized in Table 1.

**Table 1:** ICR program components.

**Components**	**SCIT, CCT, and NEAR**
Frequency	Twice a week
Duration	12 weeks
Intensity	120 min
Mode of delivery Group format	SCIT group-session, 15 min CCT strategy training, 45 min NEAR computer training Closed group
Materials	iPads and access to at least two computer programs, a whiteboard, calendars, posters, video vignettes and a device to project them, speakers to play the audio portion of video vignettes, the SCIT PowerPoint slideshows and a computer and LCD projector from which to project them
Homework Staff	Meeting with a practice partner, a staff member at the EIP service, once a week to complete exercises related to the ICR material A leading facilitator and a co-facilitator

*SCIT, Social Cognition and Interaction Training; CCT, Compensatory Cognitive Training; NEAR, Neuropsychological Educational Approach to Remediation*.

### Implementation of ICR

#### Stage 1: Exploration

A timeline for the implementation process is provided in Figure 1. At the exploration stage, research evidence supporting the need for CR in early psychosis was examined and presented by author OGV to the staff. A randomized controlled trial was designed to investigate the immediate and long-term efficacy of the intervention and feasibility. A CR program was selected, and an informal cost-analysis was conducted. The program had to meet the cognitive needs of the patients and be feasible for the EIP service. Instead of using the existing neuro- and social-cognitive interventions, we decided that integrating NEAR, CCT, and SCIT would be the best fit and contain several key elements facilitating sustainability. The intervention is group-based and relatively short (12 weeks), which may be more economically feasible than an individual-based approach or a longer treatment. Other treatment programs at the EIP service are run twice per year, in spring and fall, and ICR would fit well into that scheduling. It was also important that the computerized training would be conducted on iPads to reduce the start-up cost and space required for training, as well as to allow the group to be mobile, i.e., be conducted in different rooms at the clinic.

Cost estimates included labor and non-labor expenses. The intervention and training for the intervention would be undertaken as part of the facilitators' general role in the service, with therapy time guaranteed and case weighting altered to facilitate staff capacity to undertake this work. Non-labor costs included the cost for acquiring intervention-related material (iPads, access to computer programs, calendars and treatment manuals). Ten iPads were donated by a charitable organization, but the EIP service covered all other start-up costs and provided space and staff for the intervention.

#### Stage 2: Adoption and Installation

An implementation team was formed and OGV was identified as the implementation leader who would translate the material, coordinate assessments, and provide the intervention as part of her clinical work. The ICR team met twice a month and included two psychologists, three occupational therapists, two supportive counselors, and four master's or bachelor level psychology students. Training was provided by OGV, who had received training by authors DLR and EWT. In addition to reading the treatment manuals, facilitators were required to complete a 2-days course covering relevant topics and three online CR training courses provided by Columbia University (www.teachrecovery.com). To ensure fast and easy referrals, one of the ICR facilitators attended the weekly team meetings prior to implementation. At these team meetings, the treatment team, with the support of the ICR facilitator, would review the need of each patient within the team for ICR.

#### Stage 3: Initial Implementation

In the initial implementation stage, adjustments were made to the intervention based on the results from the research study and feedback from facilitators, the staff members that served as practice partners for participants during the research study, and participants. Results from the research study suggested ICR-associated improvements in verbal memory (Logical memory I; *p* = 0.018, *N*^2^ = 0.13), cognitive flexibility (Trails B; *p* = 0.004, *N*^2^ = 0.19), working memory (digit span working memory span; *p* = 0.014, *N*^2^ = 0.13), theory of mind (Hinting Task; *p* = 0.035, *N*^2^ = 0.10), and attributional style (Ambiguous Intentions Hostility Questionnaire; *p* = 0.025, *N*^2^ = 0.13), but not for social functioning or clinical symptoms. However, at 12-months follow-up, there were significant improvements on most neuro- and social-cognitive domains as well as in employment outcomes (9, 23). ICR was well received by participants, with 77.6% attendance rates. Most participants (93%) regarded the length of each session (2 h) as appropriate, and 79% thought that the length of the intervention (12 weeks) was appropriate. We, therefore, decided to make no changes to the length or intensity of the intervention. SCIT was rated by participants as the most useful approach (44.2%), followed by the NEAR approach (37.8%) and CCT strategies (18%). These results reinforced our belief that an integrated neuro- and social-cognitive program would better fit the complex needs of an early psychosis population than a neuro- or social-cognitive approach alone. Only 33% thought that exercises with a practice partner were helpful, and 43% would have preferred to have no practice partner exercises at all. However, we decided to keep them as part of ICR, as other research has established the importance of transfer techniques in enhancing generalization to everyday life (24).

ICR facilitators participated in two focus group sessions, at mid-treatment and after treatment. They were asked open questions with general prompts regarding experience with computer programs, session content, the intervention delivery, as well as the time and practicality of the intervention. The facilitators reported a lack of understanding of the purpose of each computer game and how to link material from each approach (SCIT, CCT, and the computer games) to the participant's goals. The training program was modified to include more training and reading material on this subject. Facilitators mentioned that some participants were tired after about 30 min of computer games and did not want to train any longer. We decided to discuss this issue with group members and reached a consensus that staying for 45 min was optimal, but participants would try to notice when they were getting tired and then take breaks more often. Furthermore, facilitators would reinforce the use of CCT strategies for attention/vigilance in these situations.

Practice partners were staff members and participated in one focus group session after treatment. The average completion rate for the practice partner exercises during the research study was 63%. The practice partners reported forgetting to meet with participants. We therefore added to the ICR protocol a weekly e-mail reminder to practice partners, that also included information on the content of each session. The practice partners also thought it was difficult to help participants complete exercises where they needed to come up with their own examples. More concrete examples were therefore added to the practice partner manual.

#### Stage 4: Full Implementation

Following the initial implementation, ICR was accepted as part of expected care at the EIP service. We presented the rationale for fully implementing ICR at the EIP service to clinical directors and staff at the EIP service, as well as the chief managers of the psychiatric departments at LUH. Other advantages of implementing the intervention were also presented. These included routine access to cognitive assessments and the staff's learning and applying the ICR strategies in their work with patients. The maintenance of the intervention was also discussed, including such topics as therapists' training, training for trainers, funding, and fidelity checks.

## Results

### Maintenance and Sustainability of ICR

Since the first ICR groups were conducted in 2016, ICR has been running twice a year since the fall of 2016. Sustainability outcomes are shown in Table 2. Ongoing organizational and financial support from LUH was secured. The EIP service would continue to provide program facilitators to deliver ICR as part of their clinical work and space to run the groups. LUH would cover all other costs, including purchasing iPads and access to online computer programs.

**Table 2:** Maintenance and sustainability outcomes of ICR.

**Outcome**	**Total**
Groups conducted	11
Patients treated	109
Facilitators trained	8

### Key Factors Affecting the Implementation of ICR According to Clinical Directors

**Figure 2:**
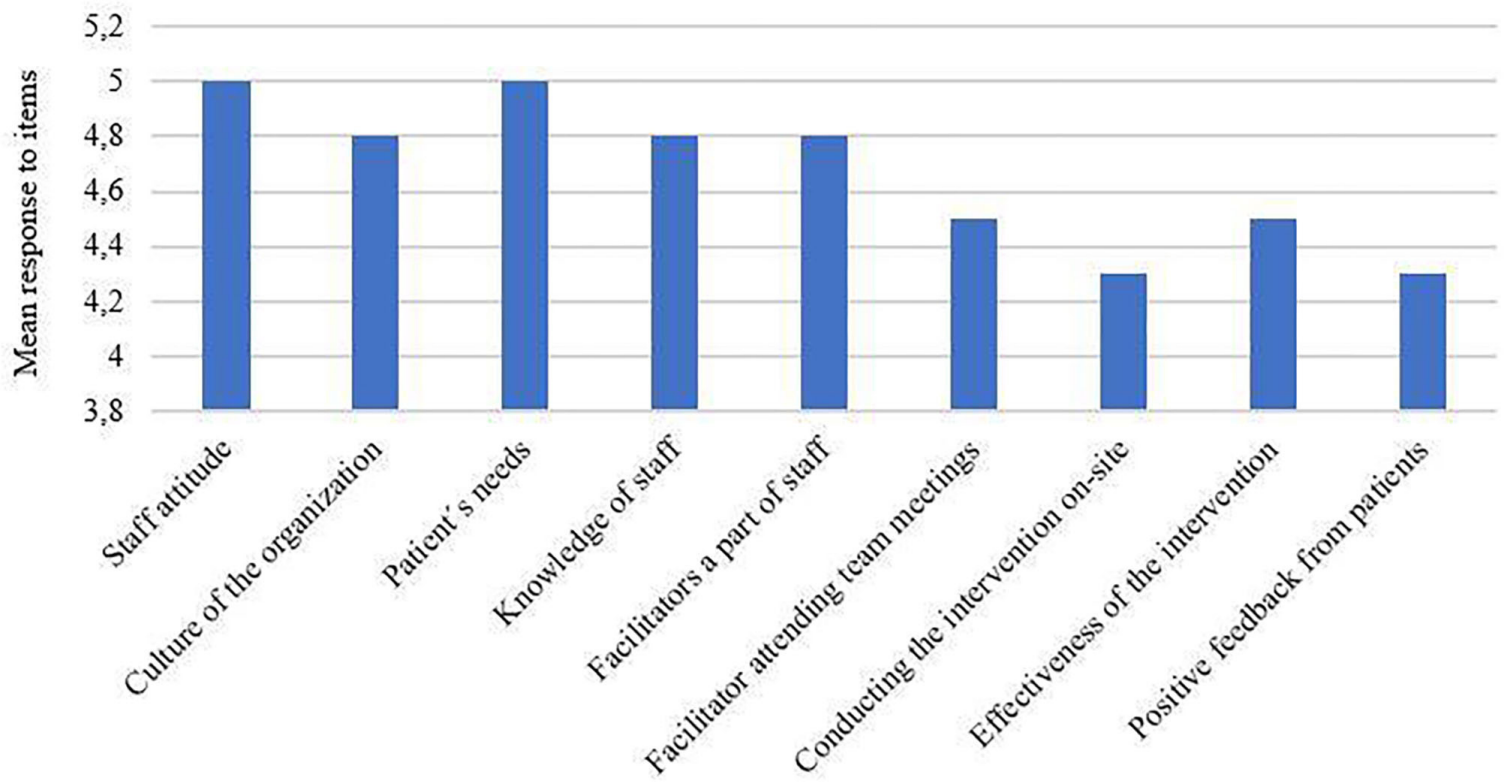
Clinical director's mean response to factors affecting the implementation process.

The results from the survey are shown in Figure 2. On average, the most important factors were staff attitude toward implementation and the patient's needs (cognitive dysfunction in the patient population). The factors scoring lowest were conducting the intervention on-site and positive feedback from patients.

The directors also commented further on some of their answers. One clinical director thought that educating the staff on the rationale behind the intervention prior to the initial implementation helped facilitate a positive attitude toward the implementation. This was important to the successful implementation because “staff members were more willing and engaged in helping clients with attendance and motivation.” Regarding perceived effectiveness, one clinical director commented: “It was important that the intervention included different approaches and could potentially benefit most, if not all, patients, whether it was by improving their cognition or social skills…. It was great to see how the patients gained more confidence in their cognitive and social abilities during and after the intervention.” The clinical directors generally thought that having EIP staff that attended team meetings as facilitators was important because “they were able to quickly inform the patient's team if attendance was dropping, as well as how the patient was performing in the intervention.” Conducting the intervention on-site was not rated as highly important by all clinical directors. One thought that conducting the intervention on-site was highly important because “it provided for a safe and familiar environment for the patients,” whereas another clinical director did not find this as important as long as the ICR facilitator attended team meetings to “give feedback to the patient's treatment team.”

## Discussion

This paper describes the implementation of ICR into a multicomponent EIP service in Iceland. ICR has become an integral sustainable component of the EIP service, with over 100 patients treated since 2016. The full implementation process took 37 months. Although this timeline is consistent with the 2- to 4-years project plan required for most such implementation projects (25), it is quite long. The implementation process could be shortened by ensuring funding and educating staff earlier in the process. In addition, by not conducting a research study, the implementation process could be shortened significantly. However, the advantages of doing a research study while implementing the treatment would be lost. Also, this would not be optimal when implementing novel treatments such as ICR.

Perhaps the most profound lesson is the importance of developing a positive attitude among clinical directors and staff toward implementation of new treatments such as ICR. Staff attitudes toward evidence-based practices have been found to be a common barrier to implementation. We took several steps throughout the implementation process to get the whole staff “on board” and to enhance their enthusiasm about the intervention. OGV held several lectures at different stages of the implementation process to educate the staff on cognitive dysfunction in the patient population, potential benefits of the intervention, the staff role in the implementation process, results from the research study, as well as feedback from patients.

Incorporating the intervention into the EIP service by including staff as facilitators and practice partners and to conduct the intervention on-site may also be important factors. Being involved in the intervention empowered them with more strategies they could apply with and teach their patients. Generalizing the strategies used in ICR may be of value. For example, there was a general need at the EIP clinic to educate both the staff and the patients on effective compensatory strategies to help patients with cognitive dysfunction with treatment adherence. It may be interesting to investigate whether the staff at the EIP service is more positive and open to new treatments that further enhance the service than staff at other psychiatric units at LUH. A flexible, open-minded attitude may be a requirement for clinical directors and staff at the multi-component and highly individualized CSC, making this setting optimal for implementation of evidence-based treatments. It may be that conducting a research study as part of the implementation process may have aided in the successful implementation of ICR, as has been demonstrated previously (18).

### Lessons Learned and Tips for Success

- Educate clinical directors and staff on the rationale for the intervention prior to implementation.
- Decide on an acceptable timeline for implementation and adjust the process accordingly.
- Conduct an on-site research study on the acceptability and effectiveness of the intervention as part of the implementation process.
- The EIP service should invest in ICR by allowing a staff member to serve as ICR team leader and oversee the referral process, training of therapists, and conducting the treatment.
- An ICR facilitator should attend team meetings at the EIP service to help identify patients that could benefit from ICR and give feedback on how participants are performing.
- Choose a program that fits the EIP service regarding length and cost. Allow for modifications of the intervention so that it is user-friendly and interoperable with the EIP service.

### Limitations

This report describes the implementation process in a particular service setting and the results may therefore not generalize to other services. These results are particularly relevant to EIP services using a CSC model. Although cognitive remediation is often promoted as cost saving in the long run, a formal cost-effectiveness analysis was not conducted.

## Conclusion

The underlying premise of this study was that access to ICR should be available routinely to all patients diagnosed with first episode psychosis in Iceland. The successful implementation and integration of ICR into the only EIP service in the country gives hope that this goal may be realized. As we move forward with implementing ICR into CSC programs, it is important to reflect on experience as well as evidence. As EIP services work to provide evidence-based and individualized care to improve the functional outcomes of their patients, they should consider implementing and integrating CR and social-cognitive training into their standard care. Further evaluation of the ICR program and dissemination to other clinics is an important next step for informing the potential systematic integration of ICR in other settings and for other patient groups.

## Data Availability Statement

The raw data supporting the conclusions of this article will be made available by the authors, without undue reservation.

## Ethics Statement

Ethical review and approval was not required for the study on human participants in accordance with the local legislation and institutional requirements. Written informed consent for participation was not required for this study in accordance with the national legislation and the institutional requirements.

## Author Contributions

OV was involved in the study design and writing the manuscript. BM, ET, and DR were involved in the study design and editing the manuscript. ES and BG were involved in editing the manuscript. All authors contributed to the article and approved the final manuscript.

## Conflict of Interest

The authors declare that the research was conducted in the absence of any commercial or financial relationships that could be construed as a potential conflict of interest.

## References

[1] Andrew A Knapp M McCrone P Parsonage M Trachtenberg M Effective Interventions in Schizophrenia: The Economic Case. Personal Social Services Research Unit, London School of Economics and Political Science, London, United Kingdom (2012).

[2] Bilder RM. Neuropsychology of first-episode schizophrenia: initial characterization and clinical correlates. Am J Psychiatry. (2000) 157:549–59. 10.1176/appi.ajp.157.4.549 10739413

[3] Green MF Bearden CE Cannon TD Fiske AP Hellemann GS Horan WP. Social cognition in schizophrenia, part 1: performance across phase of illness. Schizophr Bull. (2012) 38:854–64. 10.1093/schbul/sbq171 21345917 PMC3406534

[4] Bowie CR Harvey PD. Cognitive deficits and functional outcome in schizophrenia. Neuropsychiatr Dis Treat. (2006) 2:531–6. 10.2147/nedt.2006.2.4.531 19412501 PMC2671937

[5] Vidarsdottir OG Twamley EW Roberts DL Gudmundsdottir B Sigurdsson E Magnusdottir BB. Social and non-social measures of cognition for predicting self-reported and informant-reported functional outcomes in early psychosis. Scand J Psychol. (2019) 60:295–303. 10.1111/sjop.12549 31111499

[6] Green MF Robert SK Braff DL Mintz J. Neurocognitive deficits and functional outcome in schizophrenia: are we measuring the “right stuff”? Schizophr Bull. (2000) 26:119–36. 10.1093/oxfordjournals.schbul.a033430 10755673

[7] McGurk SR Twamley EW Sitzer DI McHugo GJ Mueser KT. A meta-analysis of cognitive remediation in schizophrenia. Am J Psychiatry. (2007) 164:1791–802. 10.1176/appi.ajp.2007.07060906 18056233 PMC3634703

[8] Wykes T Huddy V Cellard C McGurk SR Czobor P. A meta-analysis of cognitive remediation for schizophrenia: methodology and effect sizes. Am J Psychiatry. (2011) 168:472–85. 10.1176/appi.ajp.2010.10060855 21406461

[9] Vidarsdottir OG Roberts DL Twamley EW Gudmundsdottir B Sigurdsson E Magnusdottir BB. Integrative cognitive remediation for early psychosis: results from a randomized controlled trial. Psychiatry Res. (2019) 273:690–8. 10.1016/j.psychres.2019.02.007 31207854

[10] Galletly C Castle D Dark F Humberstone V Jablensky A Killackey E. Royal Australian and New Zealand College of Psychiatrists clinical practice guidelines for the management of schizophrenia and related disorders. Aust N Z J Psychiatry. (2016) 50:410–72. 10.1177/0004867416641195 27106681

[11] SIGN Scottish Intercollegiate Guidelines Network (SIGN): Management of schizophrenia (publication no. 131) SIGN, Edinburgh (2013). Available online at: https://www.gov.uk/government/uploads/system/uploads/ attachment_data/file/213761/dh_124058.pdf (accessed June 4, 2019).

[12] Verma S Chan LL Chee KS Chen H Chin SA Chong SA. Ministry of Health clinical practice guidelines: schizophrenia. Singapore Med J. (2011) 52:521–5. 21808964

[13] Vinogradov S. Has the time come for cognitive remediation in schizophrenia…again? Am J Psychiatry. (2019) 176:262–4. 10.1176/appi.ajp.2019.19020160 30929502

[14] Wyatt RJ. Neuroleptics and the natural course of schizophrenia. Schizophr Bull. (1991) 17:325–51. 10.1093/schbul/17.2.325 1679255

[15] Correll CU Galling B Pawar A Krivko A Bonetto C Ruggeri M. Comparison of early intervention services vs treatment as usual for early-phase psychosis: a systematic review, meta-analysis, and meta-regression. JAMA Psychiatry. (2018) 75:555. 10.1001/jamapsychiatry.2018.0623 29800949 PMC6137532

[16] Bilder RM Reiter G Bates J Lencz T Szeszko P Goldman RS. Cognitive development in schizophrenia: follow-back from the first episode. J Clin Exp Neuropsychol. (2006) 28:270–82. 10.1080/13803390500360554 16484098

[17] Heinssen RK Goldstein AB Azrin ST Evidence-Based Treatments for First Episode Psychosis: Components of Coordinated Specialty Care. Bethesda, MD: National Institute of Mental Health (2014).

[18] Medalia A Erlich MD Soumet-Leman C Saperstein AM. Translating cognitive behavioral interventions from bench to bedside: the feasibility and acceptability of cognitive remediation in research as compared to clinical settings. Schizophr Res. (2019) 203:49–54. 10.1016/j.schres.2017.07.044 28768601 PMC5790637

[19] Dark F Implementation and dissemination of evidence-based mental health practices. In: Medalia A, Bowie CR, editors. Cognitive Remediation to Improve Functional Outcomes. New York, NY: Oxford University Press (2016). p. 117–37.

[20] Medalia A Herlands T. Saperstein AM Revheim N Cognitive Remediation for Psychological Disorders: Therapist Guide (Second edition). New York, NY: Oxford University Press (2017).

[21] Twamley EW Vella L Burton CZ Heaton RK Jeste DV. Compensatory cognitive training for psychosis: effects in a randomized controlled trial. J Clin Psychiatry. (2012) 73:1212–9. 10.4088/JCP.12m07686 22939029 PMC3593661

[22] Roberts DL Penn DL Combs DR Social Cognition and Interaction Training (SCIT): Treatment Manual. New York, NY: Oxford University Press (2016).

[23] Vidarsdottir OG Twamley EW Roberts DL Sigurdsson E Gudmundsdottir B Magnusdottir BB. Integrative cognitive remediation for early psychosis: a 12-month follow-up. Psychiatry Res. (2020) 288:112964. 10.1016/j.psychres.2020.112964 32361338

[24] Tas C Danaci AE Cubukcuoglu Z Brüne M. Impact of family involvement on social cognition training in clinically stable outpatients with schizophrenia - a randomized pilot study. Psychiatry Res. (2012) 195:32–8. 10.1016/j.psychres.2011.07.031 21831453

[25] Fixsen D Naoom S Blase K Friedman R Wallace F. Implementation Research: A Synthesis of the Literature. Tampa, FL: University of South Florida, Louis de la parte Florida mental health institude, National implementation Research Network (FMHI Publication #231) (2005).

